# A New Cu/Fe Layer Double Hydroxide Nanocomposite Exerts Anticancer Effects against PC-3 Cells by Inducing Cell Cycle Arrest and Apoptosis

**DOI:** 10.3390/biomedicines11092386

**Published:** 2023-08-25

**Authors:** Mohamed Y. Zaky, Rehab Mahmoud, Ahmed A. Farghali, Hany Abd El-Raheem, Ahmed Hassaballa, Mohamed Mohany, Dalal Hussien M. Alkhalifah, Wael N. Hozzein, Abdelrahman Mohamed

**Affiliations:** 1 Molecular Physiology Division, Zoology Department, Faculty of Science, Beni-Suef University, Beni-Suef 62511, Egypt; 2 UPMC Hillman Cancer Center, Division of Hematology and Oncology, Department of Medicine, University of Pittsburgh, Pittsburgh, PA 15213, USA; 3 Chemistry Department, Faculty of Science, Beni-Suef University, Beni-Suef 62511, Egypt; radwaraft@yahoo.com; 4 Materials Science and Nanotechnology Department, Faculty of Postgraduate Studies for Advanced Science (PSAS), Beni-Suef University, Beni-Suef 62511, Egypt; ahmedfarghali74@yahoo.com (A.A.F.); v-habdelreheem@zewailcity.edu.eg (H.A.E.-R.); 5 Environmental Engineering Program, Zewail City of Science and Technology, October Gardens, Giza 12578, Egypt; 6 Nutrition and Food Science, College of Liberal Arts and Sciences, Wayne State University, Detroit, MI 48202, USA; eu3412@wayne.edu; 7 ZeroHarm L.C., Farmington Hills, Farmington, MI 48333, USA; 8 Department of Pharmacology and Toxicology, College of Pharmacy, King Saud University, Riyadh 11451, Saudi Arabia; mmohany@ksu.edu.sa; 9 Department of Biology, College of Science, Princess Nourah bint Abdulrahman University, Riyadh 11671, Saudi Arabia; dhalkalifah@pnu.edu.sa; 10 Botany and Microbiology Department, Faculty of Science, Beni-Suef University, Beni-Suef 62511, Egypt; hozzein29@yahoo.com

**Keywords:** Cu/Fe LDH, prostate cancer, apoptosis, cell cycle arrest

## Abstract

Prostate cancer treatment poses significant challenges due to its varying aggressiveness, potential for metastasis, and the complexity of treatment options. Balancing the effectiveness of therapies, minimizing side effects, and personalizing treatment strategies are ongoing challenges in managing this disease. Significant advances in the use of nanotechnology for the treatment of prostate cancer with high specificity, sensitivity, and efficacy have recently been made. This study aimed to synthesize and characterize a novel Cu/Fe layer double hydroxide (LDH) nanocomposite for use as an anticancer agent to treat prostate cancer. Cu/Fe LDH nanocomposites with a molar ratio of 5:1 were developed using a simple co-precipitation approach. FT-IR, XRD, SEM, TEM, TGA, and zeta potential analyses confirmed the nanocomposite. Moreover, the MTT cell viability assay, scratch assay, and flow cytometry were utilized to examine the prospective anticancer potential of Cu/Fe LDH on a prostate cancer (PC-3) cell line. We found that Cu/Fe LDH reduced cell viability, inhibited cell migration, induced G_1_/S phase cell cycle arrest, and triggered apoptotic effect in prostate cancer cells. The findings also indicated that generating reactive oxygen species (ROS) formation could improve the biological activity of Cu/Fe LDH. Additionally, Cu/Fe LDH showed a good safety impact on the normal lung fibroblast cell line (WI-38). Collectively, these findings demonstrate that the Cu/Fe LDH nanocomposite exhibited significant anticancer activities against PC-3 cells and, hence, could be used as a promising strategy in prostate cancer treatment.

## 1. Introduction

Prostate cancer is the most frequently diagnosed form of cancer for men in the US and Europe [1,2]. Prostate, colon, and lung cancer represent approximately 42% of the overall new cases reported in men, with prostate cancer reporting alone one out of every five new diagnoses [3]. Since the number of patients suffering from prostate cancer is increasing alarmingly, finding a sure-shot remedy to help increase life expectancy as well as reduce mortality rates is extremely crucial. Several cancer therapies, such as surgery, chemotherapy, radiotherapy, gene therapy, and immunotherapy, have emerged over the years [4]. Each of these methods has its own set of drawbacks, including ineffective tumor removal, poor target specificity, and severe side effects [5]. Hence, providing innovative cancer therapeutic approaches has become a global priority.

The development of modern curative nanomedicine has fueled research efforts to investigate and develop multifunctional biomaterial nanosystems that meet the strict requirements of clinical medicine [6,7,8]. This interdisciplinary field has also enabled innovative therapeutic modalities for cancer, wherein the rapid evolution of nanomaterials is the vital foundation and prerequisite for assessing the final therapeutic effect [9,10].

Organic-based nanostructures have thoroughly been studied in nanomedicine, with clinically significant family members approaching the clinical stage. Inorganic nanomaterials, on the other side, have primarily been developed over the past decade, with remarkable advances in recent years. Such inorganic nanomaterials possess desired and specific structures, compositions, morphologies, and characteristics that conventional organic nanosystems lack [11,12,13,14]. Two-dimensional (2D) nanoparticles are a commonly used type of nanomaterials with distinctive characteristics, including a large surface area and particular physical–chemical features resulting from their two-dimensional morphologies and ultra-thin thickness [15]. These characteristics endorse their application as effective components in the treatment of cancer.

Layered double hydroxides (LDHs) have received significant attention in the two-dimensional nanosheet family. LDHs are anionic clays that resemble brucite and have the chemical formula [M(II)_(1−x)_ M(III)_x−_ (OH)_2_]_x+_ (A^n−^)_x/n_.yH_2_O [16]. M (II) is a divalent cation, similar to Ni, Zn, or Cu. M (III) is a trivalent cation, similar to Al, Fe, or Ga.; A^n−^ is an LDH composed of an upper shell of positively charged metal hydroxide and an inner part of negatively charged inorganic or organic ionic species to balance the overall charge [17]. Owing to their good biocompatibility, biodegradability, ion exchange capacities, ease of surface treatment, as well as their chemical stability, LDH-based nanomaterials have attracted the attention of many researchers in the medical research field [18,19,20]. These features qualify the use of LDH for countering microbes [21], inflammation, fungi [22], wound healing [23], drug delivery, drug release [24], and cancer treatment [25]. LDHs react with H_2_O_2_ in the hypoxic environment of the tumor, which changes their structure and causes the drug to be released, increasing therapeutic efficacy. In the future, LDHs augmented with nanoparticles might be utilized in a lower pH medium for quick and precise cancer target medication delivery [26]. Even though excellent work has been undertaken, only a few efforts have promoted the further advance of LDH-based nanocomposites in prostate cancer therapy.

Many biomedical researchers are interested in copper-based nanomaterials (Cu-based NMs), due to their excellent biocompatibility and distinctive characteristics. One of the materials in cancer treatment that has been investigated the most is cu-based NMs. Biomedicine has advanced significantly in recent years, particularly in the treatment and diagnosis of cancers [27]. Cu-based NMs have received excessive interest in the nanomedicine field owing to their distinct physical–chemical characteristics and good biocompatibility. Furthermore, compared to other nanomaterials, they are easier to tune their features such as structure, surface characteristics, and size during the preparation process, lending themselves to diverse biomedical applications [28]. Most Cu-based materials have been researched, under the hypothesis that Cu ions are less toxic to healthy cells than they are to cancer cells [29]. The antitumor activity of Cu/Fe LDH nanocomposites in prostate cancer has not been extensively studied. Hence, this study aimed to examine the anticancer properties of the developed Cu/Fe LDH nanocomposite against PC-3 cells in an effort to advance an effective chemotherapeutic agent.

## 2. Materials and Methods

### 2.1. Chemicals

For the synthesis of LDH, copper nitrate (Cu (NO_3_)_2_.3H_2_O) and iron nitrate (Fe (NO_3_)_3_.9H_2_O) from WINLAB and ALPHA CHEMIKA, India, respectively, were used. Hydrochloric acid and sodium hydroxide were obtained from Biochem-Egypt. Double-distilled water was used to make all of the solutions.

### 2.2. Synthesis of the Binary Cu/Fe LDH

Cu/Fe LDH was synthesized as follows: 100 mL aqueous solution containing Cu^2+^: Fe^3+^ in a molar ratio of 5:1 was placed in a beaker with vigorous stirring. A 2 M solution of NaOH was added dropwise until the value of pH reached 10. The formed precipitates were left during 24 h at 70 °C. The precipitates obtained were repeatedly filtered as well as thoroughly washed with distilled water. Finally, the wet precipitates formed were allowed to dry overnight at 80 °C. To attain a homogeneous particle size, the LDH was then ground.

### 2.3. Characterization of the Prepared LDH

Different tools were applied to characterize the prepared nanocomposite. The crystallinity and structural composition of the obtained Cu/Fe LDH were examined using XRD with Cu-Kα radiation (wavelength is 0.154 nm) at a current and voltage of 35 mA and 40 kV, respectively; the rate of scanning was 8° min^−1^ from 5° to 80° (2θ). The prepared material (0.50 mg) was homogenized with about 300 mg optically high-purity KBr and vacuumed for 5 min, then hard-pressed for 15 min at pressure of about 10 ton.cm^−2^ until the area of KBr pellet reached 1.13 cm^2^ (Zeiss standard). The infrared spectra of a light-grey transparent pellet with no visible grains were noted on a spectrometer (Bruker Vertex 70, Germany); the range of wave numbers was between 400 and 4000 cm^−1^. To examine the morphological characteristics of Cu/Fe LDH, a high-resolution transmission electron microscope (HR-TEM) (JEM2100/Japan) and a field-emission scanning electron microscope (SEM) (JSM5610LA/Japan) were used. TGA/DTA was applied with SDT Q600 V20.9 Build 20 under N_2_ gas and at 10 °C/min rate of heating. The zeta potential of dispersed Cu/Fe LDH was measured using dynamic light scattering (Malvern, UK) to assess colloidal stability. Variations in the surface charge have been monitored in water dispersant with an index of refraction of 1.330.

### 2.4. Cell Culture

The Holding Company for Biological Products and Vaccines (VACSERA), which is located in Egypt, provided the human prostate cancer cell line PC-3 and the human normal lung fibroblast cell line (WI-38) to the research. PC-3 cell line was cultured in RPMI 1640 medium (Gibco; San Jose, CA, USA), while WI-38 was cultured in DMEM medium (Gibco; San Jose, CA, USA), supplemented with 10% fetal bovine serum. The cells were incubated at 37 °C in a 5% CO_2_ humidified incubator. Upon reaching confluency, the cells were detached using trypsin-EDTA (Invitrogen, Thermo Fisher Scientific, Inc., Waltham, MA, USA). Trypan blue dye, 3-(4,5-dimethylthiazol-2-yl)-2,5-diphenyltetrazolium bromide (MTT), as well as dimethyl sulfoxide (DMSO) were all purchased from Sigma (St. Louis, MO, USA). All of the following were purchased from Lonza, Belgium: foetal bovine serum, buffer solution (4-(2-hydroxyethyl)-1-piperazineethanesulfonic acid), L-glutamine, gentamicin, 0.25% trypsin–ethylenediaminetetraacetic acid, osmium tetroxide, phosphate-buffered saline (PBS), sodium cacodylate buffer, uranyl acetate stain, lead citrate, acetone, 4% glutaraldehyde, and 10% formalin.

### 2.5. MTT Assay

PC-3 and WI-38 cells were cultured in 96-well plates at 2 × 10^6^ cells/well. After 24 h, they were treated with Cu/Fe LDH at different concentrations: 500, 250, 125, 62.5, 31.25, 15.8, 7.9, 3.9, 2, and 1 μg/mL. Control cells were treated with DMSO. MTT was provided to each well after 48 h of drug treatment for another 2 h of incubation. The formazan created was dissolved in DMSO, and the optical density (OD) at 590 nm was used to calculate the percentage viability (Sunrise, TECAN, Inc., Morrisville, NC, USA). The viability percentage was determined as [1 − (ODt/ODc)] × 100%, where ODt is the mean optical density of test sample-treated wells and ODc is the mean optical density of untreated cells. GraphPad Prism software (San Diego, CA, USA), Version 5, was used to estimate the IC50% for each conc. using dose response curve graphic plots [30].

### 2.6. Scratch Assay

In 6-well plates, PC-3 cells were grown to full confluence before being gently scratched by drawing a straight line on the surface of the well with a sterile 200-μL pipette tip. To eliminate exfoliated cells, the cells were washed with PBS. Cu/Fe LDH (33.39 μg/mL) was applied to adherent cells and cultured for 0, 24, or 48 h. Cell migration during recovery was monitored under a microscope (Olympus, Tokyo, Japan).

### 2.7. Cell Cycle and Apoptosis Assay

Cu/Fe LDH-treated PC-3 cells were washed, fixed in 70% ethanol overnight at 4 °C, collected and centrifuged, and subjected to flow cytometry analysis (BD FACSCalibur, Eysins, Switzerland) on a FACSCalibur system [31]. Approximately 1 million cells/mL were stained with propidium iodide (PI) to determine the cell cycle distribution. Cells were stained with Annexin V-FITC and labeled with PI to determine apoptotic cell populations, as the manufacturer had instructed.

### 2.8. ROS Production Assay

In the PC-3 control wells, 0.1 mL of sample/standard dilution buffer was introduced. The Cu/Fe LDH treated wells received 0.1 mL of diluted sample each, followed by adding 0.1 mL of the detection antibody working solution. These wells were then covered with a new adhesive strip and incubated at 37 °C for 2 h with shaking at 400 rpm. Afterward, the plate was kept at 37 °C for 90 m under a cover. A sheet of absorbent filter paper was positioned on top of the plate. Subsequently, the wells were loaded with 0.1 mL of biotin detection antibody working solution. The plate was again covered and incubated at 37 °C for 60 m and then washed thrice with wash buffer. Next, each well received 0.1 mL of streptavidin-biotin complex working solution and was covered for a 30 m incubation at 37 °C. After removing the cover, the plate was thoroughly washed with the wash buffer. Following this, each well was treated with 90 mL of 3,3,5,5-tetramethylbenzidine substrate, and the plate was incubated in darkness at 37 °C for 15–30 mi. The first few wells displayed blue shades, while the remaining wells remained colorless. To complete the procedure, 50 mL of stop solution was added to each well, inducing a color change from blue to yellow. The optical density (OD) was then measured at 450 nm using a microplate reader [32].

### 2.9. Statistics

All results were displayed as mean ± standard error of the mean (SEM) with three biological repetitive experiments. GraphPad Prism software (version 5.01) was used to perform two Student’s *t*-tests, with *p* values less than 0.05 indicating significant differences.

## 3. Results and Discussion

### 3.1. Cu/Fe LDH Characterization

**Figure 1:**
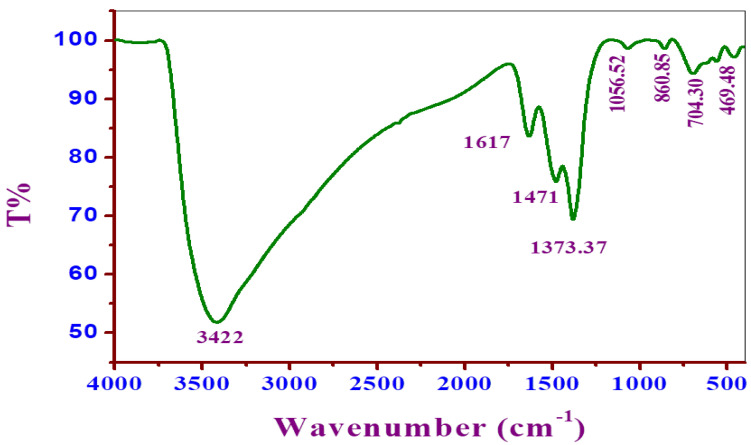
Fourier transform infrared spectrum of Cu/Fe LDH.

The FT-IR spectrum of Cu/Fe LDH exhibited a prominent wide band at 3422 cm^−1^, that may have been caused mostly by O–H stretching vibration modes of the water molecules physically adsorbed in addition to the hydrogen bonding –OH present in the interlamellar groups. (Figure 1). At 1617 cm^−1^, the bending vibration was observed. The peak band observed at 1373.37 cm^−1^, corresponding to the NO_3_ group’s stretching vibration in the LDH interlayer [33,34]. The peak bands from 469.48–860.85 cm^−1^ could be attributed to asymmetric stretching in M–O as well as M–O–H bonds, in accordance with a recent study [22].

**Figure 2:**
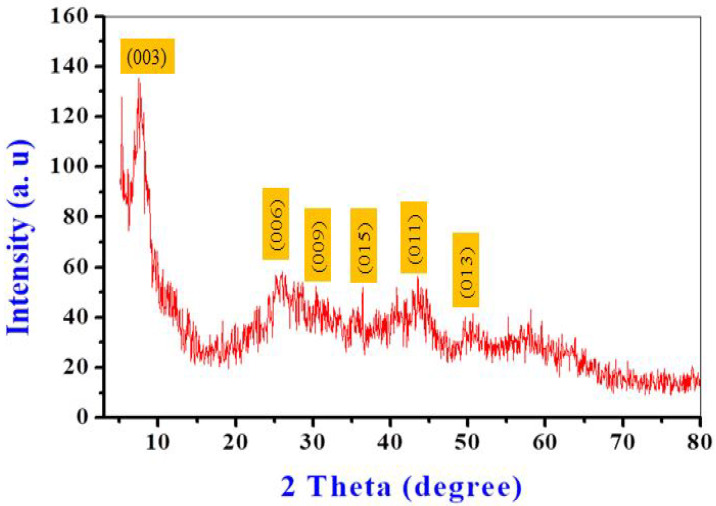
X-ray diffraction spectra of the prepared Cu/Fe LDH.

The crystalline structure and configuration of the obtained nanocomposites were interpreted using XRD analysis (Figure 2). The XRD patterns of the obtained materials were compared to those of hydrotalcite-like LDH materials. The XRD pattern of the prepared material showed a series of peaks at regular intervals, corresponding to the stacking periodicity of a layered structure [35]. The presence of the distinctive reflections of LDHs, with basal planes of (003), (006) peaks at low 2θ angles, and other peaks for (009), (015), (018), (110), (113), which correspond to planes at high 2θ angles 7.61°, 25.33°, 32.32°, 40.23°, 43.89°, 50.55°, and 57.63°, were observed, along with a rhombohedral structure (JCPDS card No. 50-0235) [30,36]. According to the XRD analysis, the basal spacing is 11.61 Å for the highest peak intensity at 2θ = 7.61° [37].

**Figure 3:**
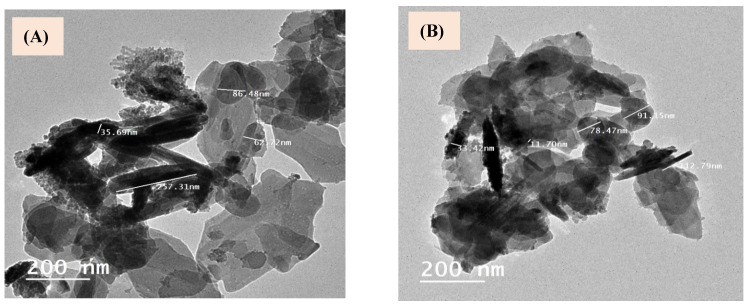
Transmission electron micrographs (**A**,**B**) of Cu/Fe LDH.

With the aid of a high-resolution transmission electron microscopy (HR-TEM), we thoroughly checked the microstructure as well as morphology of the produced LDHs, and the captured images of the Cu/Fe LDH sample are shown here (Figure 3). This microscopic method showed the formation of nanosheets with regular shapes (Figure 3A,B). The HR-TEM images revealed well-crystallized particles, with sizes ranging from 33 to 91 nm and a hierarchical plate-like structure with the typical features of an LDH crystallite.

**Figure 4:**
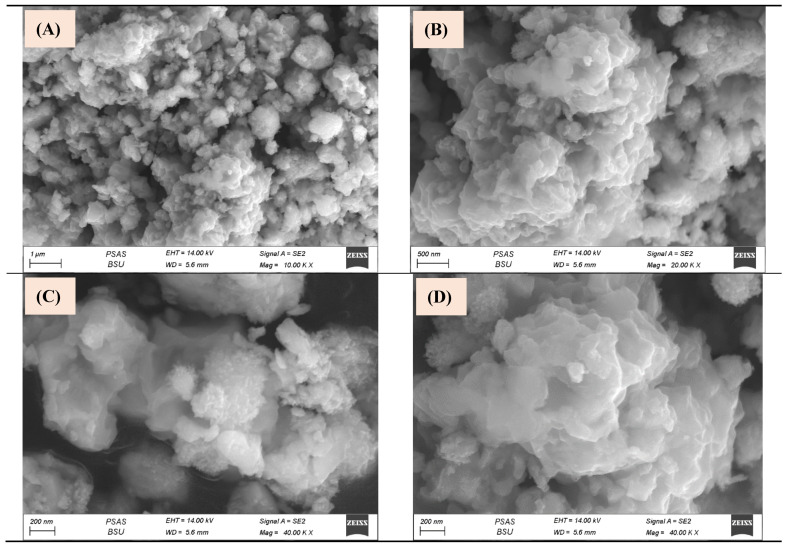
Scanning electron micrographs (**A**–**D**) of the prepared Cu/Fe LDH.

A scanning electron microscope (SEM) was applied to study particles’ morphology. The presented images of Cu/Fe LDH were captured at various magnifications, 1 µm, 500 nm, as well as at 200 nm (Figure 4). These images revealed a highly homogeneous/rough surface with several layers of the synthesized Cu/Fe LDH (Figure 4A–D). Obviously, all designed LDHs were agglomerated and possessed a flower-like morphology located on top of one another [38].

**Figure 5:**
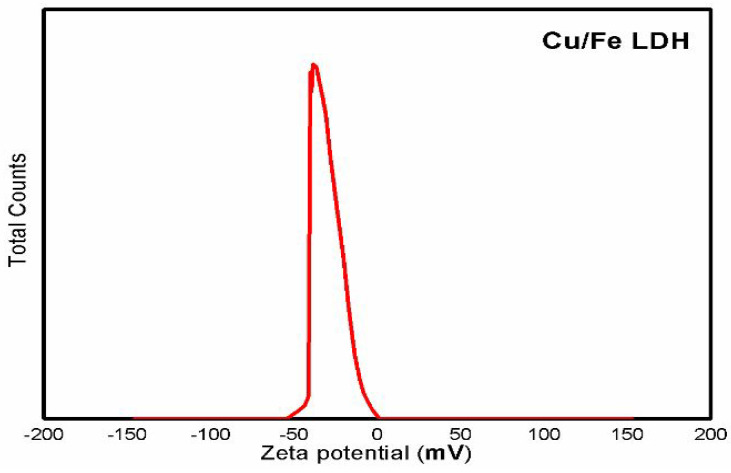
Zeta potential of Cu/Fe LDH nanoparticle suspension.

The values of zeta potential could provide information about the stability of the produced nanoparticles [39]. A zeta potential at ±30 mV is considered optimum for good stabilization of a nanodispersion [40]. The value of the zeta potential of the prepared particles was found to be −25.4 mV, indicating greater stability in aqueous solutions (Figure 5). This also implies that the nanoparticles repelled each other and did not flocculate [41,42].

**Figure 6:**
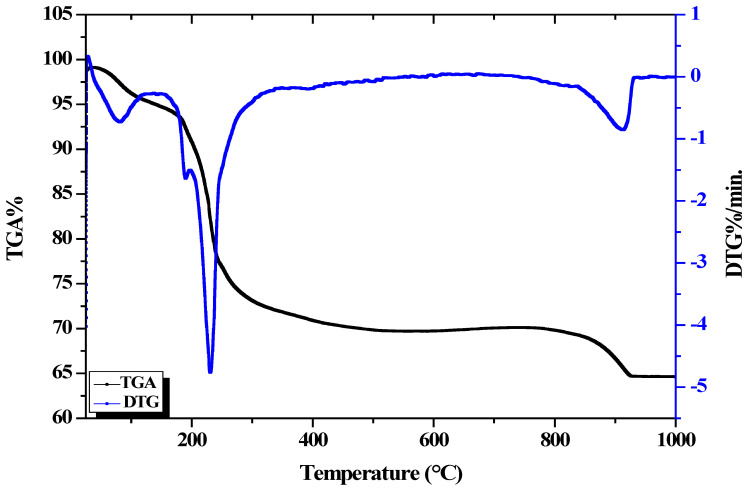
Thermogravimetric data of the synthesized Cu/Fe LDH.

A TGA of the Cu/Fe LDH was applied to investigate the thermal behavior of these nanoparticles (Figure 6). The thermal stability of the prepared LDH was determined using TGA/DTA. We found that the dehydration step occurred within the 80–250 °C temperature range. Moreover, mass changes were negligible between 400 °C and 800 °C for the hybrid material. The dehydroxylation of the LDH was completed as the temperature of the analysis increased, and an amorphous oxide phase was formed, followed by the formation of crystalline oxide phases. The final weight loss stage was caused by anionic nitrate decomposition within the layers and brucite dehydration [43].

### 3.2. Effect of the Cu/Fe LDH on PC-3 and WI-38 Cell Viability

**Figure 7:**
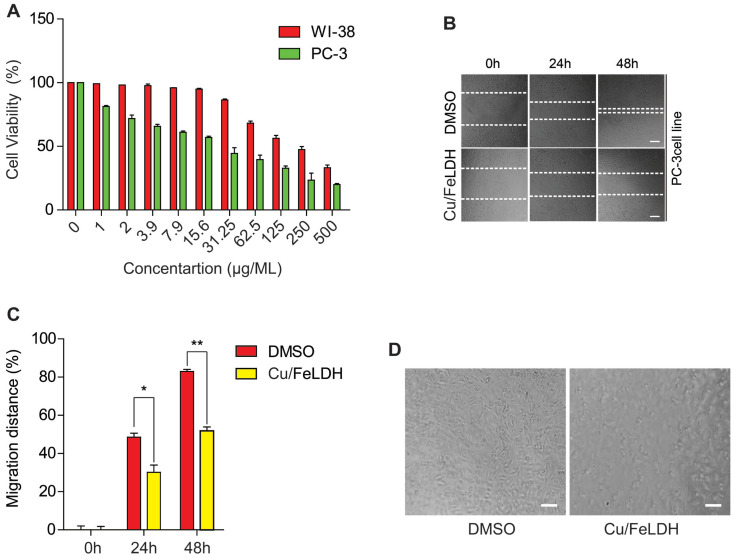
(**A**) PC-3 and WI-38 cells were treated with Cu/Fe LDH (500, 250, 125, 62.5, 31.25, 15.8, 7.9, 3.9, 2, and 1 μg/mL) for 48 h before MTT assay was conducted to estimate the cell viability. (**B**,**C**) Scratch assays were performed, wherein PC-3 cells were treated with Cu/Fe LDH (33.39 μg/mL) or with DMSO (control). The wound area’s demonstrative micrographs were collected at the indicated time points and are presented now with scale bars of 50 µm. The average distance migrated by PC-3 cells is depicted in column charts. (**D**) Morphological changes in PC-3 cells after Cu/Fe LDH treatment. Data are presented as mean ± standard error of the mean, with * and ** indicating *p* < 0.05 and *p* < 0.01, respectively.

The MTT assay was employed to assess cell viability after 48 h of treatment with Cu/Fe LDH in PC-3 and WI-38 cell lines (Figure 7A). In general, the vitality of PC3 cells decreased as the Cu/Fe LDH concentration increased, and it decreased sharply at high concentrations. The half-maximal inhibitory concentration (IC50%) against PC-3 was 33.39 μg/mL, which shows that the Cu/Fe LDH nanocomposite has a strong cytotoxic effect against PC-3 cells. Moreover, to investigate the potential safety of the Cu/Fe LDH nanocomposite towards normal cells, the cytotoxic activity of the Cu/Fe LDH towards the human normal lung fibroblast cell line (WI-38) was investigated. The results showed that Cu/Fe LDH exhibited a good safety impact on WI-38 normal cells. The nanocomposite’s IC50% against WI-38 was 222.7 μg/mL, indicating that it has an excellent safety impact as an anticancer agent. Thus, we established that the Cu/Fe LDH nanocomposite exhibits anticancer activity against PC-3 cells by inhibiting their growth, which is in line with previous reports [25,44]. LDH offers many distinct advantages as an anticancer agent due to its uniform distribution of metal ions and the facile exchangeability of intercalated anions. LDH, and its nanocomposites can be promising candidates for prostate cancer therapy.

### 3.3. Effect of the Cu/Fe LDH on PC-3 Migration

Using a scratch assay, we investigated the effect of Cu/Fe LDH on PC-3 cell migration. DMSO-treated control PC-3 cells recovered by 48% and 82%, on average, after 24 and 48 h, respectively, whereas cells treated with Cu/Fe LDH recovered by 26% and 42%, on average, after the same time points (Figure 7B,C). Further, we assessed the influence of the nanocomposite on the morphology of PC-3 cells after treating them with 33.39 µg/mL of Cu/Fe LDH for 24 h. DMSO-treated cells appeared normal and were well adhered to the surface. However, Cu/Fe LDH-treated cells exhibited significantly reduced adhesion capacity, cell size, and cell density (Figure 7D), suggesting that the Cu/Fe LDH nanocomposite exerts potent anticancer activity against PC-3 cells by inhibiting their migration and altering their morphology.

### 3.4. Effect of the Cu/Fe LDH on PC-3 Cell Cycle Arrest and Apoptosis

**Figure 8:**
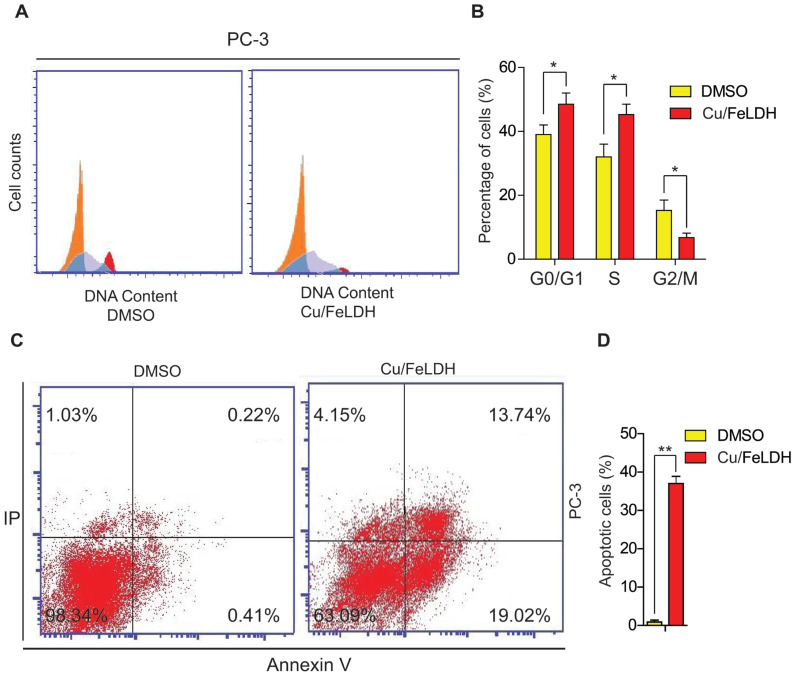
(**A**) The cell cycle distribution of PC-3 cells using flow cytometry. (**B**) Column charts show the G_1_, S, and G_2_/M PC-3 cell stages. PC-3 cells were treated with Cu/Fe LDH (33.39 μg/mL), and the control group was treated with DMSO for 24 h. (**C**) PC-3 cells were treated with Cu/Fe LDH (33.39 μg/mL) for 24 h, and the control group was treated with DMSO. (**D**) The column charts quantify the percentage of apoptotic PC-3 cells. Data are presented as mean ± SEM, with * and ** indicating *p* < 0.05 and *p* < 0.01, respectively.

To investigate the antiproliferative effect of Cu/Fe LDH, we conducted a cell cycle arrest analysis after treating PC-3 cells with the nanocomposite for 24 h. Cell cycle progression is closely related to cancer development. In the control group, 38.52% of the cells were distributed in the G_1_ phase, whereas this proportion increased significantly to 57.51% in the Cu/Fe LDH-treated group (Figure 8A,B). Similarly, the proportion of PC-3 cells in the S phase significantly increased from 32.12% in the control group to 48.32% in the Cu/Fe LDH-treated group. The major checkpoints in the cell cycle are in the G_1_ and G_2_ phases, and they crucially regulate the progression of the cell cycle [45]. Based on our findings, we speculated that Cu/Fe LDH induced G_1_/S cell cycle arrest, which led to its potent antiproliferative effect against PC-3 cells. The Annexin V-FITC/PI was also used to assess the percentage of apoptosis in PC-3 cells prompted by Cu/Fe LDH. Our Results showed that Cu/Fe LDH at 33.39 µg/mL increased apoptosis by 36.91% (Figure 8C,D), confirming the proapoptotic effect of Cu/Fe LDH on PC-3 cells. Apoptosis is an essential homeostatic system in the body that sustains a specific balance between cell division and cell death as well as adequate cell numbers [46]. Reactive oxygen species, which produce undesirable redox reactions and interfere with protein and DNA functions, may be responsible for Cu/Fe LDH-mediated cytotoxicity, G_1_/S cell cycle arrest, and apoptosis. Overall, our findings show that Cu/Fe LDH exhibits antiproliferative and proapoptotic activities against PC-3 cells.

### 3.5. Effect of the Cu/Fe LDH on ROS Production

**Figure 9:**
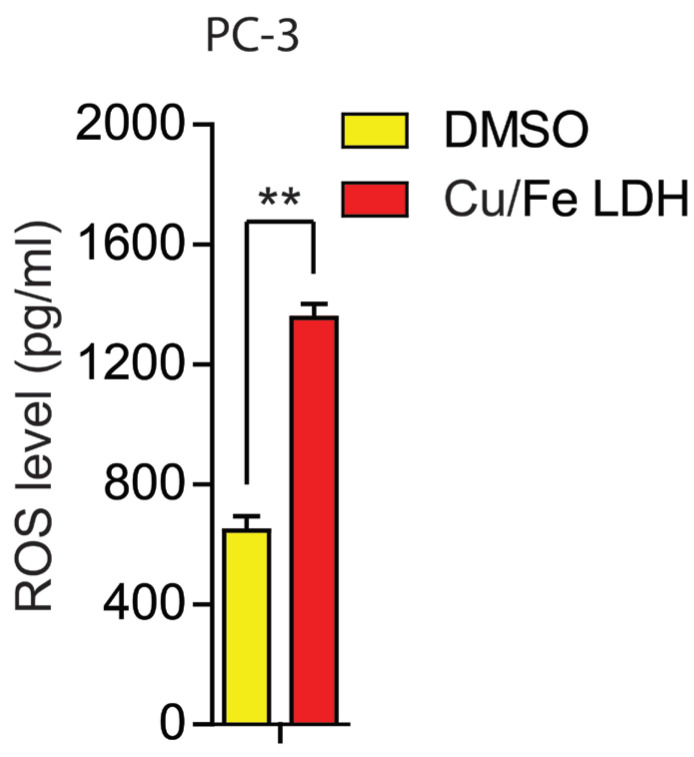
ROS production levels in PC-3 cells. PC-3 cells were treated with Cu/Fe LDH (33.39 μg/mL), and the control group was treated with DMSO for 24 h. The control group was treated with DMSO. Data are presented as mean ± SEM, with ** indicating *p* < 0.01.

ROS functions as a secondary messenger in cell signaling, regulating various biological processes in both healthy and cancerous cells [47]. Chemotherapeutics induce ROS production to trigger cell death, akin to its natural role [48]. To manage ROS fluctuations during cancer treatment, drugs enhancing apoptosis are employed [49,50]. Our study demonstrates that treating PC-3 cells with Cu/Fe LDH effectively elevates ROS levels (Figure 9), inducing apoptosis and confirming its anti-proliferative and pro-apoptotic effects. While moderate ROS production maintains cell viability, excessive ROS impairs antioxidant defenses, potentially causing oxidative stress, DNA damage, and apoptosis [51,52,53]. Thus, our study underscores ROS’s importance in PC-3 inducing apoptosis.

## 4. Conclusions

**Figure 10:**
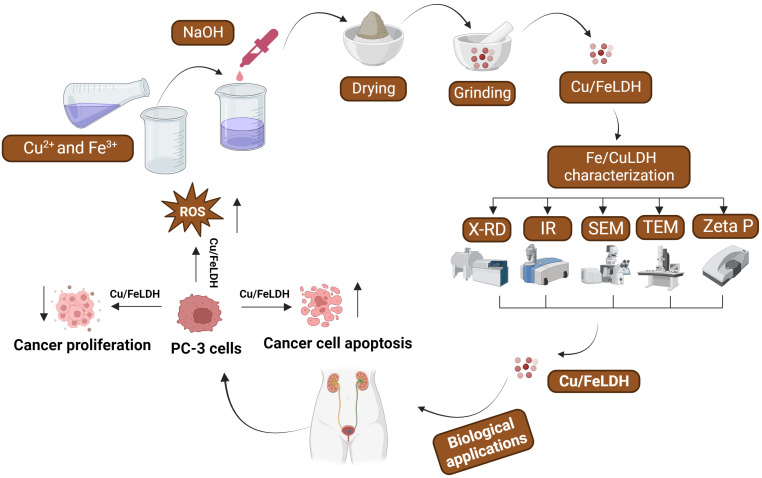
Schematic depicting the syntheses, characterizations, and anticancer activity of a novel Cu/Fe LDH nanocomposite.

In this study, a Cu/Fe LDH nanocomposite was synthesized. The obtained nanocomposite was tested and characterized using both microscopic and spectroscopic methods. We verified the potency of this nanocomposite as an anticancer agent against the prostate cancer cell line PC-3. Cell viability sharply decreased when cells were treated with a high concentration of LDH, indicating its significant cytotoxic impact on PC-3 cells. Additionally, Cu/Fe LDH showed a good safety impact on the normal lung fibroblast cell line (WI-38). Cu/Fe LDH also reduced cell migration and altered cell morphology. Furthermore, these findings offer innovative insights into how CuFe/LDH induces apoptosis in PC-3 cells. It establishes a relation between inhibiting cell growth and triggering apoptosis, and it highlights that cell death resulted from the generation of ROS and cell cycle arrest. The developed nanocomposite formula displayed antiproliferative and proapoptotic activities against PC-3 cells (Figure 10). Overall, the synthesized Cu/Fe LDH nanocomposite exhibited substantial anticancer activities against PC-3 cells and may be exploited as a promising strategy in prostate cancer treatment.

## Data Availability

All data can be found in the article.

## References

[1] Malvezzi M., Carioli G., Bertuccio P., Rosso T., Boffetta P., Levi F., La Vecchia C., Negri E. (2016). European cancer mortality predictions for the year 2016 with focus on leukaemias. Ann. Oncol..

[2] Siegel R.L., Miller K.D., Fuchs H.E., Jemal A. (2021). Cancer statistics. CA Cancer J. Clin..

[3] Zhang X., Li G., Wu D., Li X., Hu N., Chen J., Chen G., Wu Y. (2019). Recent progress in the design fabrication of metal-organic frame-works-based nanozymes and their applications to sensing and cancer therapy. Biosensors Bioelectron..

[4] Sahu A., Kwon I., Tae G. (2019). Improving cancer therapy through the nanomaterials-assisted alleviation of hypoxia. Biomaterials.

[5] Cheng L., Wang C., Feng L., Yang K., Liu Z. (2014). Functional Nanomaterials for Phototherapies of Cancer. Chem. Rev..

[6] Wang X., Zhong X., Li J., Liu Z., Cheng L. (2021). Inorganic nanomaterials with rapid clearance for biomedical applications. Chem. Soc. Rev..

[7] Zhong X., Wang X., Li J., Hu J., Cheng L., Yang X. (2021). ROS-based dynamic therapy synergy with modulating tumor cell-microenvironment mediated by inorganic nanomedicine. Coord. Chem. Rev..

[8] Zhuang Y., Han S., Fang Y., Huang H., Wu J. (2022). Multidimensional transitional metal-actuated nanoplatforms for cancer chemo-dynamic modulation. Coord. Chem. Rev..

[9] Dong C., Feng W., Xu W., Yu L., Xiang H., Chen Y., Zhou J. (2020). The Coppery Age: Copper (Cu)-Involved Nanotheranostics. Adv. Sci..

[10] Yang N., Li W., Gong F., Cheng L., Dong Z., Bai S., Xiao Z., Ni C., Liu Z. (2020). Injectable Nonmagnetic Liquid Metal for Eddy-Thermal Ablation of Tumors under Alternating Magnetic Field. Small Methods.

[11] Ren X., Huang X., Wu Q., Tan L., Fu C., Chen Y., Meng X. (2021). Nanoscale metal organic frameworks inhibition of pyruvate kinase of M2. Chin. Chem. Lett..

[12] Ruan J., Qian H. (2021). Recent Development on Controlled Synthesis of Mn-Based Nanostructures for Bioimaging and Cancer Therapy. Adv. Ther..

[13] Ravichandiran P., Jegan A., Premnath D., Periasamy V.S., Vasanthkumar S. (2015). Design, synthesis, molecular docking as histone deacetylase (HDAC8) inhibitors, cytotoxicity and antibacterial evaluation of novel 6-(4-(4-aminophenylsulfonyl) phenyla-mino)-5 H-benzo [a] phenoxazin-5-one derivatives. Med. Chem. Res..

[14] Ravichandiran P., Sivakumar A.S., SeonYoung K., Jong Soo K., Byung-Hyun P., Kwan Seob S., Dong Jin Y. (2019). Synthesis and an-ticancer evaluation of 1, 4-naphthoquinone derivatives containing a phenylaminosulfanyl moiety. ChemMedChem.

[15] Tan C., Zhang H. (2015). Epitaxial growth of hetero-nanostructures based on ultrathin two-dimensional nanosheets. J. Am. Chem. Soc..

[16] Li Y., Bi H.Y., Shi X.Q. (2015). Simultaneous adsorption of heavy metal and organic pollutant onto citrate-modified layered double hy-droxides with dodecylbenzenesulfonate. Environ. Eng. Sci..

[17] Goh K.-H., Lim T.-T., Dong Z. (2008). Application of layered double hydroxides for removal of oxyanions: A review. Water Res..

[18] Yan L., Gonca S., Zhu G., Zhang W., Chen X. (2019). Layered double hydroxide nanostructures and nanocomposites for biomedical applications. J. Mater. Chem. B.

[19] Chatterjee A., Bharadiya P., Hansora D. (2019). Layered double hydroxide based bionanocomposites. Appl. Clay Sci..

[20] Chimene D., Alge D.L., Gaharwar A.K. (2015). Two-Dimensional Nanomaterials for Biomedical Applications: Emerging Trends and Future Prospects. Adv. Mater..

[21] El-Shahawy A.A., El-Ela F.I., Mohamed N.A., Eldine Z.E., El Rouby W.M. (2018). Synthesis and evaluation of layered double hydrox-ide/doxycycline and cobalt ferrite/chitosan nanohybrid efficacy on gram positive and gram negative bacteria. Mater. Sci. Eng. C.

[22] Moaty S.A., Farghali A., Khaled R. (2016). Preparation, characterization and antimicrobial applications of Zn-Fe LDH against MRSA. Mater. Sci. Eng. C.

[23] El-Ela F.I.A., Farghali A.A., Mahmoud R.K., Mohamed N.A., Moaty S.A.A. (2019). New Approach in Ulcer Prevention and Wound Healing Treatment using Doxycycline and Amoxicillin/LDH Nanocomposites. Sci. Rep..

[24] Yasaei M., Khakbiz M., Ghasemi E., Zamanian A. (2019). Synthesis and characterization of ZnAl-NO_3_(-CO_3_) layered double hydroxide: A novel structure for intercalation and release of simvastatin. Appl. Surf. Sci..

[25] Bhattacharjee A., Rahaman S.H., Saha S., Chakraborty M., Chakraborty J. (2019). Determination of half maximal inhibitory concentration of CaAl layered double hydroxide on cancer cells and its role in the apoptotic pathway. Appl. Clay Sci..

[26] Jayakumar A., Surendranath A., Mohanan P.V. (2018). 2D materials for next generation healthcare applications. Int. J. Pharm..

[27] Zhong X., Xingliang D., Yan W., Hua W., Haisheng Q., Xianwen W. (2022). Copper-based nanomaterials for cancer theranostics. Wiley Interdiscip. Rev. Nanomed. Nanobiotechnol..

[28] Liu K., Liu K., Liu J., Ren Q., Zhao Z., Wu X., Li D., Yuan F., Ye K., Li B. (2020). Copper chalcogenide materials as photothermal agents for cancer treatment. Nanoscale.

[29] Acilan C., Cevatemre B., Adiguzel Z., Karakas D., Ulukaya E., Ribeiro N., Correia I., Pessoa J.C. (2017). Synthesis, biological characteri-zation and evaluation of molecular mechanisms of novel copper complexes as anticancer agents. Biochim. Biophys. Acta Gen. Subj..

[30] Mosmann T. (1983). Rapid colorimetric assay for cellular growth and survival: Application to proliferation and cytotoxicity assays. J. Immunol. Methods.

[31] Banjerdpongchai R., Suwannachot K., Rattanapanone V., Sripanidkulchai B. (2008). Ethanolic rhizome extract from Kaempferia par-viflora Wall. ex. Baker induces apoptosis in HL-60 cells. Asian Pac. J. Cancer Prev..

[32] Alarifi S., Ali D., Suliman A., Ahamed M., Siddiqui M.A., Al-Khedhairy A.A. (2013). Oxidative stress contributes to cobalt oxide na-noparticles-induced cytotoxicity and DNA damage in human hepatocarcinoma cells. Int. J. Nanomed..

[33] Younes H.A., Khaled R., Mahmoud H.M., Nassar H.F., Abdelrahman M.M., El-Ela F.I., Taha M. (2019). Computational and experimental studies on the efficient removal of diclofenac from water using ZnFe-layered double hydroxide as an environmentally benign absorbent. J. Taiwan Inst. Chem. Eng..

[34] Abo El-Reesh G.Y., Farghali A.A., Taha M., Mahmoud R.K. (2020). Novel synthesis of Ni/Fe layered double hydroxides using urea and glycerol and their enhanced adsorption behavior for Cr (VI) removal. Sci. Rep..

[35] Aguirre J.M., Adamo G., Oscar G. (2011). Simple route for the synthesis of copper hydroxy salts. J. Braz. Chem. Soc..

[36] Zaher A., Taha M., Farghali A.A., Mahmoud R.K. (2020). Zn/Fe LDH as a clay-like adsorbent for the removal of oxytetracycline from water: Combining experimental results and molecular simulations to understand the removal mechanism. Environ. Sci. Pollut. Res..

[37] Mahgoub S.M., Shehata M.R., Zaher A., El-Ela F.I.A., Farghali A., Amin R.M., Mahmoud R. (2022). Cellulose-based activated carbon/layered double hydroxide for efficient removal of Clarithromycin residues and efficient role in the treatment of stomach ulcers and acidity problems. Int. J. Biol. Macromol..

[38] Rodeghiero E.D., Chisaki J., Giannelis E.P. (1997). In situ microstructural control of Ni/Al2O3 and Ni/NiAl2O4 composites from layered double hydroxides. Chem. Mater..

[39] Kokila T., Ramesh P.S., Geetha D. (2015). Biosynthesis of silver nanoparticles from Cavendish banana peel extract and its antibacterial and free radical scavenging assay: A novel biological approach. Appl. Nanosci..

[40] Mikolajczyk A., Gajewicz A., Rasulev B., Schaeublin N., Maurer-Gardner E., Hussain S., Leszczynski J., Puzyn T. (2015). Zeta Potential for Metal Oxide Nanoparticles: A Predictive Model Developed by a Nano-Quantitative Structure–Property Relationship Approach. Chem. Mater..

[41] Grover I.S., Singh S., Pal B. (2013). The preparation, surface structure, zeta potential, surface charge density and photocatalytic activity of TiO_2_ nanostructures of different shapes. Appl. Surf. Sci..

[42] Pandi P., Gopinathan C. (2017). Synthesis and characterization of TiO_2_–NiO and TiO_2_–WO_3_ nanocomposites. J. Mater. Sci. Mater. Electron..

[43] Awes H., Zaki Z., Abbas S., Dessoukii H., Zaher A., Abd-El Moaty S.A., Shehata N., Farghali A., Mahmoud R.K. (2021). Removal of Cu^2+^ metal ions from water using Mg-Fe layered double hydroxide and Mg-Fe LDH/5-(3-nitrophenyllazo)-6-aminouracil nano-composite for enhancing adsorption properties. Environ. Sci. Pollut. Res..

[44] Saha S., Ray S., Ghosh S., Chakraborty J. (2018). pH-dependent facile synthesis of CaAl-layered double hydroxides and its effect on the growth inhibition of cancer cells. J. Am. Ceram. Soc..

[45] Chakravarti B., Maurya R., Siddiqui J.A., Bid H.K., Rajendran S., Yadav P.P., Konwar R. (2012). In vitro anti-breast cancer activity of ethanolic extract of Wrightia tomentosa: Role of pro-apoptotic effects of oleanolic acid and urosolic acid. J. Ethnopharmacol..

[46] You C., Han C., Wang X., Zheng Y., Li Q., Hu X., Sun H. (2012). The progress of silver nanoparticles in the antibacterial mechanism, clinical application and cytotoxicity. Mol. Biol. Rep..

[47] Sauer H., Wartenberg M., Hescheler J. (2001). Reactive Oxygen Species as Intracellular Messengers during Cell Growth and Differentiation. Cell. Physiol. Biochem..

[48] Bossis G., Sarry J.-E., Kifagi C., Ristic M., Saland E., Vergez F., Salem T., Boutzen H., Baik H., Brockly F. (2014). The ROS/SUMO Axis Contributes to the Response of Acute Myeloid Leukemia Cells to Chemotherapeutic Drugs. Cell Rep..

[49] Ivanova D., Zhelev Z., Aoki I., Bakalova R., Higashi T. (2016). Overproduction of reactive oxygen species—Obligatory or not for induction of apoptosis by anticancer drugs. Chin. J. Cancer Res..

[50] Wang X., Lu X., Zhu R., Zhang K., Li S., Chen Z., Li L. (2017). Betulinic Acid Induces Apoptosis in Differentiated PC12 Cells Via ROS-Mediated Mitochondrial Pathway. Neurochem. Res..

[51] Marchi S., Giorgi C., Suski J.M., Agnoletto C., Bononi A., Bonora M., De Marchi E., Missiroli S., Patergnani S., Poletti F. (2012). Mitochondria-Ros Crosstalk in the Control of Cell Death and Aging. J. Signal Transduct..

[52] Magnano S., Barroeta P.H., Duffy R., O’Sullivan J., Zisterer D.M. (2021). Cisplatin induces autophagy-associated apoptosis in human oral squamous cell carcinoma (OSCC) mediated in part through reactive oxygen species. Toxicol. Appl. Pharmacol..

[53] Zhang W., Zhu Y., Yu H., Liu X., Jiao B., Lu X., Libertellenone H. (2021). A Natural Pimarane Diterpenoid, Inhibits Thioredoxin System and Induces ROS-Mediated Apoptosis in Human Pancreatic Cancer Cells. Molecules.

